# The battle of Alzheimer’s Disease – the beginning of the future Unleashing the potential of academic discoveries

**DOI:** 10.3389/fphar.2014.00102

**Published:** 2014-05-09

**Authors:** Johan Lundkvist, Magnus M. Halldin, Johan Sandin, Gunnar Nordvall, Pontus Forsell, Samuel Svensson, Liselotte Jansson, Gunilla Johansson, Bengt Winblad, Jonas Ekstrand

**Affiliations:** ^1^AlzeCure Foundation, Karolinska Institutet Science Park NovumHuddinge, Sweden; ^2^Alzheimerfonden, FrösundavikSolna, Sweden; ^3^Center for Alzheimer Research at Karolinska Institutet and Swedish Brain PowerNovum, Huddinge, Sweden

**Keywords:** Alzheimer’s disease, drug discovery and development, technology, pharmaceutical, non-profit, collaborative research

## Abstract

Alzheimer’s Disease (AD) is the most common form of dementia, affecting approximately 36 million people worldwide. To date there is no preventive or curative treatment available for AD, and in absence of major progress in therapeutic development, AD manifests a concrete socioeconomic threat. The awareness of the growing problem of AD is increasing, exemplified by the recent G8 Dementia Summit, a meeting held in order to set the stage and steer the compass for the future. Simultaneously, and paradoxically, we have seen key players in the pharmaceutical industry that have recently closed or significantly decreased their R&D spending on AD and other CNS disorders. Given the pressing need for new treatments in this area, other actors need to step-in and enter this drug discovery arena complementing the industrial efforts, in order to turn biological and technological progress into novel therapeutics. In this article, we present an example of a novel drug discovery initiative that in a non-profit setting, aims to integrate with both preclinical and clinical academic groups and pharmaceutical industry to explore the therapeutic potential of new concepts in patients, using novel biology, state of the art technologies and rapid concept testing.

## INTRODUCTION

During the last 25 years the population of the world has increased from 5.3 to almost 7.2 billion people (World Population Review, 2013). While an increased standard of living has resulted in decreased mortality, a global increased awareness of healthy living in combination with major progress in several areas of medicine has also made significant contribution to the increase in world population. Indeed, the death caused by heart disease, stroke, and cancer have decreased in the USA from 68% (1980) to 53% (2010) (75 Years of Mortality in the United States, 1935–2010, 2012). In parallel with these encouraging numbers, another major medical threat is emerging which could result in a major socioeconomic chaos in the absence of medical progress. The threat is named Alzheimer’s Disease (AD). Worldwide, approximately 36 million people are diagnosed having dementia and 7.7 million new cases are discovered every year. AD is the most common cause of dementia among the elderly and may contribute up to 60–70% of all cases (World Health Organization, 2012). Currently there is no curative or preventive treatment for the patients and only a few drugs, which all provide limited symptomatic relief during a relatively short time frame, are available. Age is by far the largest risk factor for developing AD and the incidence increases exponentially from about 1% of 65-year old people to 30% of all 85-year-old people. With the current global increase in average lifespan, approximately 115 million people are estimated to be suffering from AD in 2050. Besides the tremendous suffering for the affected individuals and their close relatives, the cost for the society is estimated to be more than 200 billion USD in 2013 and 1200 billion USD by 2050 in the USA alone, a number that will put the healthcare system under enormous strain (Alzheimer’s Association, 2013; World Alzheimer Report 2013, 2014).

Awareness, at the highest political level, of the emerging threat of dementia was highlighted with the recent G8 summit on dementia held in London in December 2013. The aim of the meeting was to develop a coordinated global action on dementia, and to shape an effective international response to dementia, resulting in the communication of a declaration (G8 Dementia Summit Agreements, 2013). This declaration included; “an ambition to identify a cure, or a disease-modifying therapy, for dementia by 2025.” This will be achieved by, e.g., significantly increasing the amount spent on dementia research, increasing the number of people involved in clinical trials and studies on dementia as well as develop an international action plan for research. These efforts clearly state the sense of urgency in this field to move forward and for the research community to find a solution.

Dementia or senility as a concept or condition is a very old phenomenon, referred to in medical texts since antiquity. Today, we are aware of a large number of diseases that cause dementia but AD represents the most common one (Ballenger, 2006; Lane et al., 2012; Savonenko et al., 2012; Agis-Torres et al., 2014). A major milestone in the history of AD took place approximately 100 years ago (1907), when the German neuropsychiatrist Alois Alzheimer first described the key neuropathological hallmarks of the disease (Hardy, 2006). During the examination of brain sections derived from his patient Auguste D, a middle-aged woman who suffered and died from dementia, Dr. Alzheimer discovered so-called senile plaques and neurofibrillary tangles in the brain parenchyma. These were seminal findings, linking for the first time the form of dementia now called “Alzheimer’s disease,” to specific pathological changes within the brain. However, the recognition that dementia was a result of abnormal pathological changes distinct from normal aging, did not really gain momentum until the 1970s, with the birth of the cholinergic hypothesis. A number of studies showed that, in AD brains, there was a particular loss of markers for cholinergic function as well as basal forebrain cholinergic neurons early on in the disease pathology (Francisa et al., 1999). These discoveries fueled an intense research effort to find drugs that could target cholinergic dysfunction and today three out of the four commonly prescribed drugs for AD (donepezil, rivastigmine and galantamine) are so-called cholinesterase inhibitors (Greenberg et al., 2013). These drugs inhibit the degradation of acetylcholine in the synaptic cleft, thereby sustaining the action of acetylcholine with a subsequent improvement in cognitive function in the patient. However, the underlying neurodegenerative cascade is not affected by these treatments, and the effect is thereby limited in time due to the continuous loss of cholinergic neurons and thus acetylcholine production in the disease. Moreover, gastrointestinal side effects, relating to muscarinic receptor engagement in the gut, as well as headache and effects on heart rate are also dose-limiting factors for the patients. The fourth described drug for AD is memantine, a weak NMDA receptor antagonist, prescribed for moderate to severe AD. Similar to the acetylcholinesterase inhibitors, memantine provides symptomatic relief for a limited time period, but is not believed to alter the neurodegenerative cascade of the disease.

## ALZHEIMER’S DISEASE – NAVIGATION THROUGH COMPLEXITY

Although the progress in the drug development for AD has been limited over the last 30–40 years, significant progress has been made in understanding the mechanisms behind the development of AD, e.g., the pathology, neurobiology, and genetics (Corbette et al., 2012; Dunkel et al., 2012; Kepp, 2012; Greenberg et al., 2013). Among these finding were the discoveries of the amyloid beta peptide (Aβ) and the tau protein, the principal components of senile plaques and neurofibrillary tangles, respectively. Furthermore, researchers were also able to identify more than 200 disease causing mutations localized to three genes directly involved in Aβ generation as well as unravel some of the molecular machinery and underlying mechanisms of Aβ production. Combined, these discoveries provided a strong rationale for a better understanding of the neurotoxic role of Aβ in AD and for the development of Aβ amyloid-directed therapies. Accordingly, during the last 15 years, both active and passive vaccines targeting Aβ as well as several inhibitors and modulators targeting the aggregation or synthesis of Aβ peptide has been developed and tested in AD clinical trials. In addition, a large number of other drug candidates exhibiting a range of different non-Aβ targeting therapeutic mechanism of action have been tested clinically. These include those that are primarily aiming for a symptomatic effect by increasing synaptic activity, e.g., compounds that interact with nicotinic, histaminergic, and serotonergic receptors (Misra and Medhi, 2013). Despite that many of these drug candidates have shown promising effect in preclinical models, the results from clinical trials have been disappointing, and no new drugs after the acetylcholine esterase inhibitors and the NMDA blocker memantine have made it all the way to the market. It is well recognized that drug discovery in the AD field has been hampered by the failure of preclinical models to recapitulate some of the key features of the AD pathogenesis (Savonenko et al., 2012). The use of these models to unravel the complex features of the neurodegenerative cascade in AD, as well as translational tools in, e.g., dose prediction studies, have therefore been limited.

The reasons for the clinical failures are likely many, and differ among the drug candidates tested (Mangialasche et al., 2010; Schneider et al., 2014). Major contributors to the general lack of success include insufficient target exposure to achieve a clinically meaningful effect, that the drug was not tested long enough, safety issues due to on- and off-target pharmacology and/or that the disease context was not appropriate for the therapeutic mechanism explored (see **Box 1**). The latter stems from recent progress in biomarker research, which has shown that the AD pathological cascade start early and takes place over decades prior to symptoms onset, and where Aβ amyloidosis dominates at early stages followed by more overt and outbread neuronal dysfunction and neuronal degeneration, implicating other pathogenic drivers of disease, once the disease becomes symptomatic and progresses into severe stages of dementia (Sperling et al., 2011). Therefore, a therapy tailored for a specific component of the AD pathogenic cascade may have to be given to patients at a specific stage of the disease in order to be efficacious. The recent development of specific radiopharmaceutical diagnostic tools for PET imaging Aβ-amyloid plaque density (e.g., florbetapir and florbetaben) has been extremely useful in assessing amyloid load in patients at various stages of the disease (Herholz and Ebmeier, 2011). Thanks to these new methods with improved resolution combined with improvements in biomarker monitoring, the characterization and diagnosis of patients suspected to be suffering from AD pathogenesis have improved. Interestingly, such analyses have revealed that as many as 25–30% of the patients included in recent trials with therapeutic antibodies targeting Aβ amyloidosis were in fact negative for Aβ-amyloid in their brains (Doody et al., 2014; Salloway et al., 2014). These observations highlight the need to design trials where a lot of emphasis is put on the patient inclusion criteria in order to increase the odds to get meaningful clinical response of the therapeutic compound and/or mechanism tested.

**Box 1. Some key challenges to be addressed in the development of novel therapeutics for Alzheimer’s disease.**

Identify and link novel pathways and biomarkers to AD progression.

Improved preclinical modeling of disease relevant pathology.

Engaging multiple targets might be required (polypharmacology) and thereby using a multi-drug regimen.

Establish and conduct conclusive proof-of-concept (PoC) studies.

Use appropriate patient inclusion criteria in clinical trials.

Achieve sufficient CNS exposure/target engagement of the drug.

Establish appropriate biomarkers and/or surrogate markers for target engagement.

Establish surrogate markers for clinical effect to shortened the length of clinical trial.

Improved clinical endpoints, e.g., more sensitive and relevant cognitive measures.

Avoid safety issues due to chronic, systemic exposure of the drug.

Feedback to discovery from clinical PoC to refine hypothesis and improve research models.

Handle potential drug–drug interactions (patient population often on other medications).

## FUTURE IDEAS TOWARDS NOVEL AD THERAPEUTICS

### THE POTENTIAL OF ACADEMIC DISCOVERIES IN AD

The pharmaceutical industry (pharma) is currently under pressure from a range of different challenges in its environment, e.g., increased R&D costs, patent cliffs causing a major loss of income, high attrition of projects in the portfolio, increasing cost-constraints in the healthcare system mandating generics and parallel import, and not the least, more demanding regulatory requirements (Kola and Landis, 2004; Cuatrecasas, 2006; Schachter and Ramoni, 2007; Munos, 2009; Paul et al., 2010; Scannell et al., 2012; Slusher et al., 2013). In our view, an attractive alternative to the current way of working, as well as a way to offer a potential solution to the gaps in drug discovery and development pipelines, is to increase the research efforts in non-profit biopharmaceutical research institutions, such as universities and private research institutes, where creative and innovative science is not severely restricted by commercial objectives, but where novel hypothesis can be tried with industrial stringency in a dedicated way. These types of smaller research units could make significant contributions to the field since it is well recognized that small- and medium-sized biotechnological companies have been more successful than big pharmaceutical companies in moving candidate substances through the pipeline, and in particular, more successful in producing biological products, such as monoclonal antibodies, vaccines, and peptides (Munos, 2009). Non-profit organizations are well suited to address areas of large unmet need in rare CNS-disorders classified as orphan diseases. These diseases are, on the whole, not commercially blockbusters and, thus, research funded by philanthropists and public sources will be key to encourage work in these areas. It is also likely that certain therapeutics developed for an orphan indication may turn out to be efficacious in related disorders that share certain common features or principles of pathogenesis. The availability of clinically validated drugs is extremely valuable, and repositioning of existing drugs for novel indications opens up novel exciting possibilities to accelerate therapeutic development in complex CNS disorders such as AD.

Today when many major pharmaceutical companies are downsizing their R&D efforts in the CNS area (Alzheimer’s Drug Failure: implication for Future R&D in Neuroscience, 2012), they have a strong need to get access to outside expertise in specific scientific areas such as for the identification of novel targets, increasing the knowledge in certain target families, advancing new platform technologies for preclinical and clinical research, improving the diagnosis of disease, pharmacology and safety biomarker development, access to human cells and tissues for target validation, as well as how to perform earlier proof-of-mechanism (PoM) and proof-of-concept (PoC) studies, including clinical safety studies. Some of this will of course be possible through established collaborations between the pharmaceutical industry and third parties such as contract research organization (CRO) and academia, such an example is the Dominantly Inherited Alzheimer Network (DIAN, established in 2008), which test diagnostics and novel treatments in a specific patient population. Although many CROs now provide customers with various non-regulatory *in vitro* and *in vivo* experiments during early discovery, their main activities are regulatory-driven (in accordance with good laboratory practice (GLP) in later phases of drug development). However, we see an attractive alternative in non-profit biomedical research institutions, where scientific collaborations can be based on a close interaction rather than contracting. This collaborative network/partnership will facilitate a closer preclinical-to-clinical translation at the target, model, assay, substance, patient and clinical testing levels as well as in the development of new technologies. Innovation and progress driven in these areas by such constellations will certainly accelerate therapeutic development in AD, and thereby attract increased interest from the big pharmaceutical companies, given that the potential return of investment would be substantial.

## AlzeCure FOUNDATION – AN EXAMPLE

**FIGURE 1:**
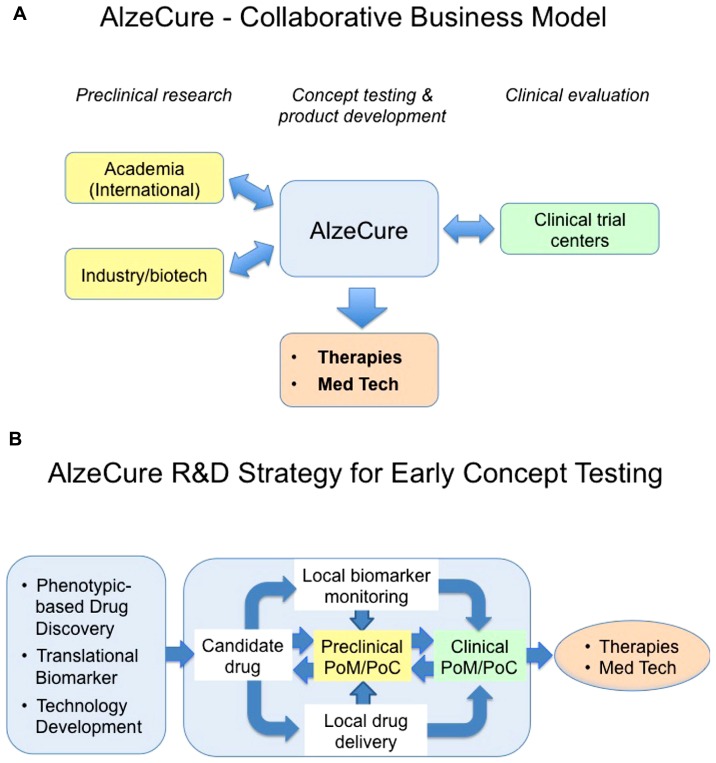
**This cartoon illustrates the proposed AlzeCure business (A) and strategy models (B) to develop new therapies and medicinal technology products, highlighting the proof-of-mechanism (PoM)/proof-of-concept (PoC) as an important milestone in drug discovery and development process.** Traditionally, in order to reach clinical PoM and PoC with “standard peripherally administered” drug candidates in a chronic condition, the drug needs to display key and optimized features including adsorption, distribution, metabolism, tolerability, and BBB permeability, in addition to its desired pharmacology and safety. This is seldom achieved despite significant medicinal chemistry, an effort that takes place over a very long time in the discovery phase. As a consequence, many suboptimal compounds are being explored in the clinic without being able to test the hypothesis they were aimed for in a conclusive manner. Building the need to get strong evidence for PoM/PoC, including close collaboration with international academic preclinical research, utilizing new Translational enabling technologies into the discovery process, is crucial. To reduce the attrition due to lack of efficacy and safety, it is important to design the first clinical trials, a design based on appropriate animal models, pharmacokinetic/pharmacodynamic modeling, and utilizing biomarkers and surrogate markers, in the “right patients,” to facilitate that the molecular target is being hit at an expected concentration to give the anticipated physiological response without safety issues. Optimally, the PoM/PoC clinical trials are conducted at Clinical Trial Centers that are closely involved in the discovery research. It is also important to have a rapid feed-back to the drug discovery process from positive or failed PoM/PoC studies to improve the understanding of mechanisms, and to accelerate the development of follow-up compounds that address the same mechanism, but with improved drug-like characteristics or to identify related mechanistic targets that will lead to new clinical studies.

AlzeCure is a Swedish-based non-profit drug discovery foundation, whose aim is to develop novel therapeutics for AD and related disorders (see **Box 2** and **Figure 1**). This new organization was founded in 2013 by former AstraZeneca scientists with more than 30 years of experience from the CNS drug discovery and development area. The team exhibits complementary skills and expertise, from medicinal chemistry, screening, *in vivo* translational/biomarker competence to ADME and clinical development. The strategy of AlzeCure Foundation (“AlzeCure”) is to use its wide experience and international network in the Alzheimer field to run collaborative projects with external expertise that has the potential to deliver real value for the patients. The team focuses on areas of research and development, which are of urgent need of innovative solutions, such as mechanistic diversity, preclinical models, drug delivery, clinical testing, and biomarker monitoring. We are convinced that rapid feedback to the drug discovery process from positive or failed PoC studies are essential in order to improve our understanding and to progress the development of efficacious therapeutics. Such backtranslation is also crucial in order to accelerate the development of follow-on compounds that address the same mechanism, but have improved drug-like characteristics, or to identify related mechanistic targets that will lead to new clinical studies. History has proven that there is a synergy between technology and biology innovation and in that interface, exciting discoveries take place that can result in novel products with a remarkable potential. Similarly, we strongly believe that technology breakthroughs will contribute significantly to speed up and improve the development of novel therapies in major CNS disorders.

**Box 2. Strategies of the AlzeCure foundation.**

Strong focus on both innovative drug discovery and clinical testing.

Use unique technology for drug administration.

Develop novel technology for biomarker monitoring.

Explore new therapeutic mechanisms and principles.

Develop of combination treatments affecting more than one biological pathway.

Test old marketed drugs or early closed candidate drugs on novel AD-targets.

Run phenotypic screens with well-defined pathological endpoints.

Collaborate with academic and industrial expertise to complement in-house expertise and capabilities.

Design early PoM and PoC clinical studies to test new hypothesis.

Optimize patient selection for earlier clinical safety and efficacy studies, based on qualified clinical biomarkers.

The laboratories of AlzeCure are located in direct vicinity to biotech companies and Karolinska Institute Alzheimer Research Center with academic groups of leading experts in neurobiology, preclinical and clinical laboratories with a long track record in AD translational research and in clinical trials. The strategic localization of AlzeCure has resulted in face-to-face interactions with these institutions and disciplines on a regular basis, which is a strong enabling factor for effective project progression and success. The non-profit nature of AlzeCure has turned-out to facilitate the establishment of joint efforts with both academia and industry and attract alternative funding. Today, AlzeCure manages several collaborations with international academic experts in both preclinical and clinical research. AlzeCure collaborates with several biotech companies, which provide unique chemistry and technology that in combination with neuronal screening assays based on induced pluripotent stem cells (iPS) and phenotypic endpoints, provide the basis for novel drug discovery. The most advanced collaboration aims at a novel way of administering therapeutic tau-directed antibodies to the brain, in order to increase target engagement and thereby therapeutic activity^1^. Tau is emerging as a new important molecular target in AD, and several anti-tau antibodies are rapidly approaching clinical trials. However, a major challenge with peripheral administration of antibodies is to reach the target in the brain to a sufficient extent. AlzeCure is together with NsGene, a Denmark and US-based biotechnology company, and Peter Davies (Feinstein Institute) evaluating a new concept to deliver therapeutic antibodies to the brain, using a novel proprietary Brain-Repair technology platform developed by NsGene (Lindvall and Wahlberg, 2008). The Brain-Repair platform is an implant comprised a semi-permeable hollow fiber containing a human cell line, which is engineered to locally produce therapeutic proteins of choice that diffuse into the target tissue. The implant has been clinically validated in patients diagnosed with AD through collaboration between NsGene and the Karolinska Institute (Eriksdotter-Jönhagen et al., 2012). An opportunity with this device would be to more rapidly test the hypothesis in a PoC study in a well-defined tauopathy patient population for an orphan indication, before entering large expensive phase III trials. In this manner, we foresee getting both the technology, therapeutic mode of action, clinical effect, and safety evaluated, in an effective and conclusive manner within a shorter time frame compared to common standards of clinical testing in AD. Obviously the Brain-Repair implant also offers a very exciting concept to rapidly test other therapeutic hypothesis beyond tau-directed antibodies, and illustrates a concrete example where technology innovation together with new biology form a novel avenue for therapy development applicable for multiple CNS disorders.

A co-initiator and also major financial contributor to AlzeCure is the Swedish Alzheimer foundation, a charity organization. This type of direct financial commitment and interest in drug discovery and development in complex disorders such as CNS diseases from charity organizations is novel and may point to a new path for future organizations. In addition, the Swedish Brain Power program is sponsoring AlzeCure with two Ph.D. positions, which are highly integrated into the academic labs thereby providing the basis for a strong two-way communication between academic science and industrial development, which is of high mutual interest. Given the declining interest from many big pharmaceutical companies and venture capitalists alike in CNS disorders and the pressing need for new medications, initiatives like these are new ways of trying to directly stimulate drug discovery efforts in an area of great medical need. Interestingly, in line with the intention of AlzeCure, Alzheimer Research UK announced at the end of 2013 that they were to initiate a Drug Discovery Institute consisting of leading academic groups in the UK that will have close access to both preclinical and clinical research units and hospitals specialized in neurodegenerative diseases that cause dementia. 

## CONCLUSION

The problem we as a society are facing is of unseen proportions, highlighting the need of concerted actions at multiple levels of society. A firm political leadership paired with a well-designed regulatory framework and strong incentives for academia, the industry and the health care providers is needed in order to consolidate the resources and optimize the process required to make rapid and significant progress in therapeutic development for AD. In this light it may seem paradoxical that several major industrial actors, such as AstraZeneca, Bristol-Myers Squibb, GlaxoSmithKline, and Novartis have either abandoned the neuroscience therapeutic indication as such, or heavily decreased their head count within this area (Abbot, 2011). However, other companies, e.g., Eli Lilly, Roche, Pfizer have continued a strong interest in the area and are closely collaborating the regulatory agencies, which recently resulted in a Guidance for Industry from FDA on Alzheimer’s disease: Developing drugs for the treatment of early stage disease (U.S. Food and Drug Administration, 2013). The medical need is however still unmet and rapidly growing, thus something radical needs to be done. For a start, we believe that new initiatives such as the AlzeCure Foundation and Drug Discovery Alzheimer Research UK group, both being non-profit and taking full advantage of the open-minded academic style of working with the stringent and goal-oriented industrial R&D, have the promise of being more innovative and effective unleashing the potential of academic discoveries and providing an exciting new framework for the long-term objective to deliver effective therapies to the benefit of the patients.

## Conflict of Interest Statement

The authors declare that the research was conducted in the absence of any commercial or financial relationships that could be construed as a potential conflict of interest.

## Footnotes

1 See http://www.alzecure.org
